# Supplementary figures and images for: Rediscovery of Laura’s glassfrog Nymphargus laurae (Anura: Centrolenidae) with new data on its morphology, colouration, phylogenetic position and conservation in Ecuador
Source: PeerJ. 2021 Dec 23;9:e12644. doi: 10.7717/peerj.12644 (PMC8710250; doi:10.7717/peerj.12644)

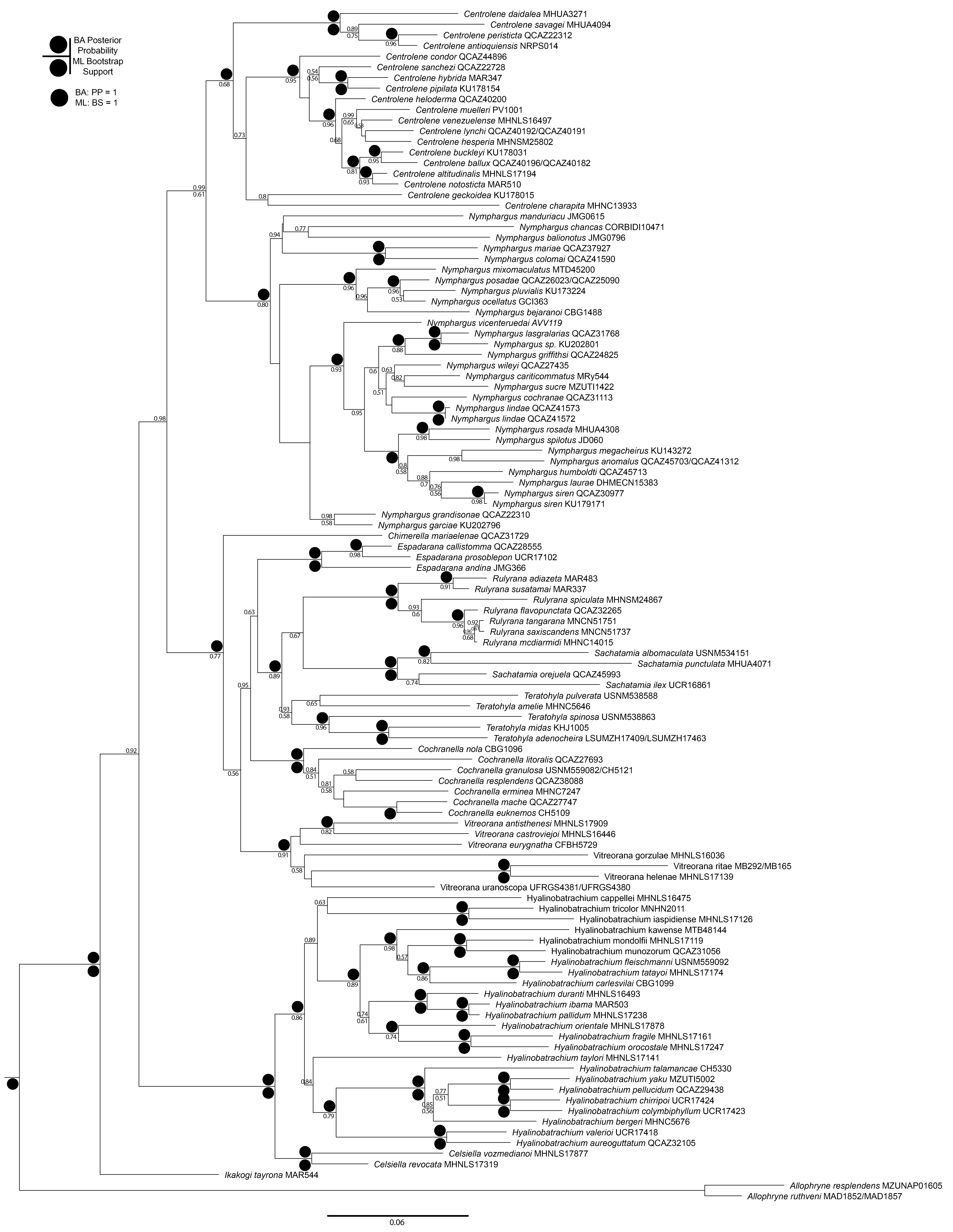

Supplement: Supplemental Information 1 — Values above nodes are posterior probabilities resulting from Bayesian phylogenetic analyses (values < 0.9 not shown, black circles = 1). Numbers bellow nodes correspond to non-parametric bootstraps (values < 0.70 not shown, black circles = 1). [file peerj-09-12644-s001.png]
